# A Case Study and Review of Literature of Eruptive Syringoma in a Six-Year-Old

**DOI:** 10.7759/cureus.14634

**Published:** 2021-04-22

**Authors:** Arthur M Samia, Deepak Donthi, Joseph Nenow, Preeti Malik, Karyn Prenshaw

**Affiliations:** 1 Department of Pathology and Laboratory Medicine, Brody School of Medicine/East Carolina University, Greenville, USA; 2 Department of Pathology and Laboratory Medicine, Vidant Medical Center/East Carolina University, Greenville, USA; 3 Public Health, Icahn School of Medicine at Mount Sinai, New York, USA; 4 Neurology, Massachusetts General Hospital, Boston, USA

**Keywords:** eruptive, syringoma, eccrine duct

## Abstract

Syringomas are benign tumors originating from the intraepidermal portion of eccrine sweat ducts. A six-year-old African American female presented with multiple 2-3 mm hyperpigmented papules over the neck, upper chest, and axillae bilaterally. The lesions were non-tender, non-pruritic, and did not bleed when lightly scraped. A café-au-lait macule was incidentally found in the mid-back of the patient. Histopathologically, multiple small ducts displaying a tadpole-shaped/paisley-tie pattern with fibrotic stroma were identified on hematoxylin and eosin staining. Epithelium showing nests of cells with basaloid appearance and dilated glands filled with eosinophilic material were also identified. These histopathologic findings were consistent with a diagnosis of eruptive syringoma. The patient was treated conservatively, and the lesions subsided without intervention. In most patients requesting treatment, isotretinoin is used; however, this may be an unnecessary measure in many patients. Overall, this case was significant due to the patient’s young age, ethnicity, and clinical improvement in the absence of treatment.

## Introduction

Syringomas are benign tumors originating from the intraepidermal portion of eccrine sweat ducts. Common sites of presentation are the periorbital area, neck, anterior chest, upper abdomen, periumbilical region, and axillae [1]. Eruptive syringomas were first described by Jacquet and Darier [2]. A classification proposed by Friedman and Butler divided them into the local form, disseminated form, inherited form, and syringoma associated with Down Syndrome [3]. Additionally, syringomas have been associated with diabetes mellitus. The eruptive syringoma subtype has been proposed to be a presentation of the generalized/disseminated form of syringomas [3]. The histogenesis of syringomas is most likely related to eccrine elements or pluripotential stem cells; however, due to histological resemblance, distinguishing eccrine versus apocrine ducts remains difficult [4]. Thus, many tumors characterized as eccrine have actually been apocrine in differentiation. The immunohistochemical pattern of cytokeratin expression in eccrine ducts indicates differentiation in both the superficial dermal duct and the deep intraepidermal duct (e.g., sweat duct ridge). Few authors believe eruptive syringoma represents a hyperplastic response of the eccrine duct to an inflammatory reaction rather than a true adnexal neoplasm [5,6].

## Case presentation

**Figure 1:**
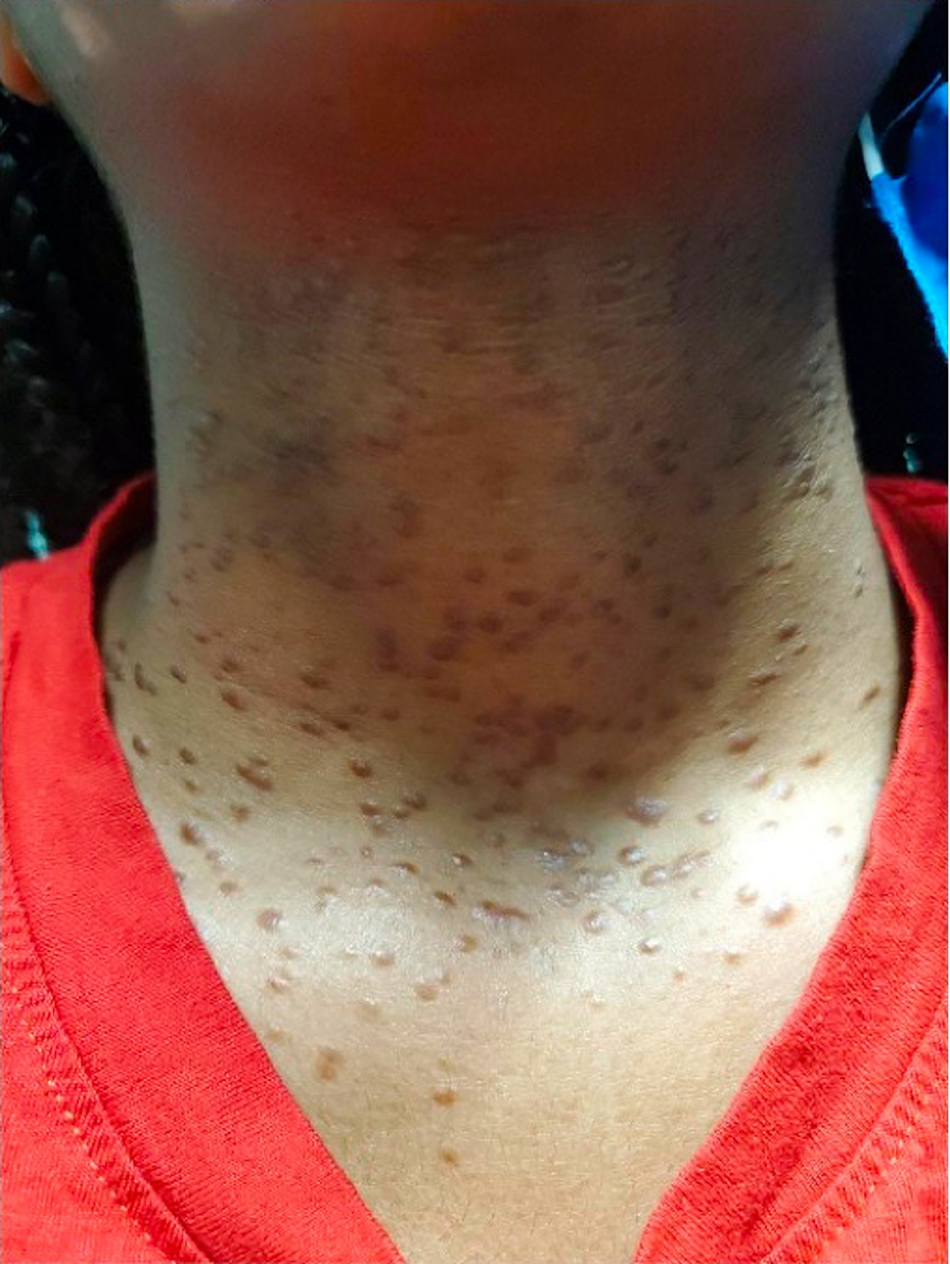
The 2-3 mm hyperpigmented papules over the patient’s neck and upper chest.

**Figure 2:**
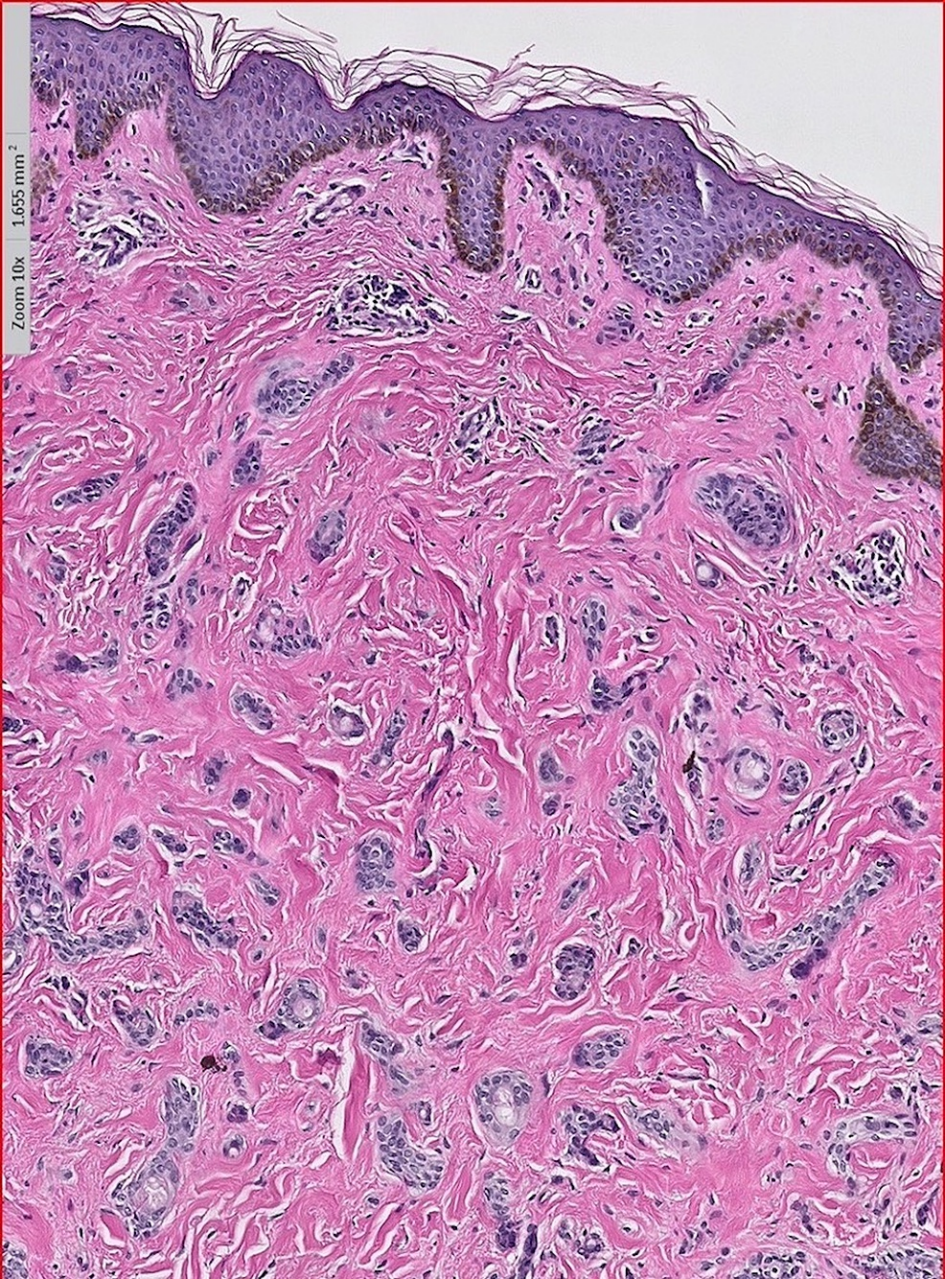
Low power (10×) revealing multiple small ducts which are tadpole-shaped/paisley-tie pattern-shaped with dense, red, sclerotic, or fibrotic stroma.

**Figure 3:**
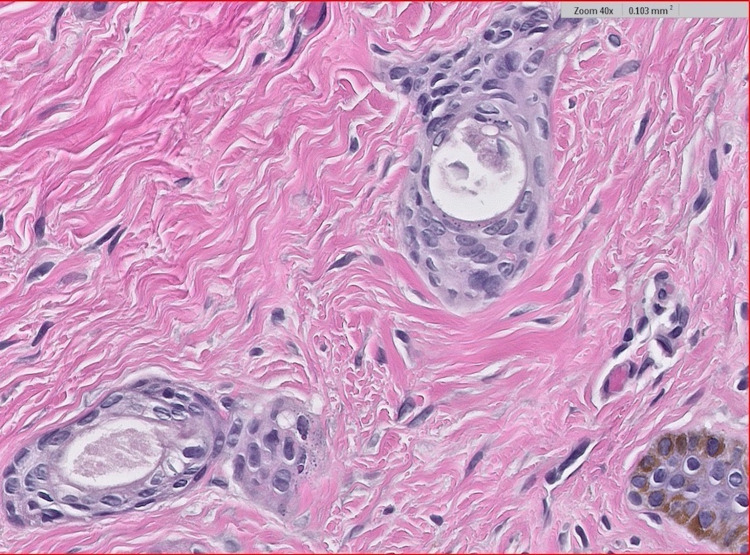
High power (40×) revealing small ducts with two layers of epithelium which have nests and strands of cells with a basaloid appearance. Dilated glands with eosinophilic material are also present.

A six-year-old African American female presented to the dermatology clinic with several 2-3 mm hyperpigmented papules that began under the chin and upper neck. The lesions were non-tender, non-pruritic, and did not bleed when lightly scraped. In the months following the initial presentation to her dermatologist, she developed an expansive eruption of similar papules over the neck, upper chest, and axillae bilaterally (Figure 1). There was also the incidental finding of a café-au-lait macule on the patient’s mid-back. Physical examination was otherwise unremarkable. One lesion from the patient’s upper chest was biopsied. Histopathological examination showed multiple small ducts displaying a tadpole-shaped/paisley-tie pattern (Figure 2). Dense, red, sclerotic, or fibrotic stroma was identified on hematoxylin and eosin staining. The epithelium was characterized by nests and strands of cells with a basaloid appearance in two distinct, thin layers. Dilated glands with lumens filled with eosinophilic material were also identified (Figure 3). The patient’s clinical picture coupled with the histopathologic findings was consistent with the lesions being syringomas. She was diagnosed accordingly, reassured that her lesions were benign, and treated conservatively due to her age. No other family members were similarly affected, and all syndromic associations were ruled out. After a three-year follow-up, the lesions decreased in size without medical or surgical intervention and were no longer increasing in quantity.

## Discussion

Eruptive syringoma is a benign adnexal neoplasm that mimics many inflammatory and malignant tumors. African Americans and Asians are known to have a higher incidence of syringomas [7,8]. Our case was of a six-year-old girl, which is an uncommon age for eruptive syringoma. However, one study reported the ages of these cases can range from 5 to 63 years [9]. Additionally, eruptive syringoma is more commonly found in females than males [10].

Clinically, these lesions are present symmetrically, bilaterally, and are known to be shiny, angulated, flat on the top, papular, and involve follicular and non-follicular areas [10]. Few studies have reported these lesions to be hyperpigmented, erythematous, Darier signs positive, or present with pseudo-koebnerization [11,12]. Frequently, the papules were present on the periorbital area, neck, anterior chest, upper abdomen, periumbilical region, and axillae [1]. They typically itch, spare the palms and soles, and are devoid of mucosal involvement [10]. The differential diagnosis for this condition based on clinical evaluation includes acne vulgaris, sebaceous hyperplasia, eruptive xanthoma, Darier’s disease, Fox-Fordyce disease, verruca plana, lichen planus, mastocytosis, granuloma annulare, lichen nitidus, pseudoxanthoma elasticum, trichoepithelioma, and sarcoidosis [13]. Histopathologically, eruptive syringomas are lined by two layers of epithelial cells with ductal dilatation to form cystic structures and few ducts may show a comma-shaped appearance of the epithelial cells [14].

Treatment for eruptive syringoma is mainly for cosmetic purposes, as there is no known long-term morbidity or mortality associated with this condition [11]. With a multitude of treatment options available, the main aim of treatment is to decrease scarring and reduce recurrence [11]. The various treatment modalities available include dermabrasion, various methods of excision, cryosurgery, electrodesiccation, chemical peeling, oral and topical retinoids, carbon dioxide laser therapy, and topical atropine [11]. However, the results are often unsatisfactory [13]. Oral isotretinoin is the most commonly used treatment modality due to its systemic effect on these lesions; however, the risk of recurrence is unaffected [11]. One study exhibited moderately effective treatment results by using a Q-switched alexandrite laser and subsequent temporary tattooing [15]. In general, surgical approaches are less useful due to the widespread nature of these lesions [11].

Of the 42 cases of eruptive syringoma evaluated in the literature, 12 patients completed one or more treatment courses and the remaining 30 were managed conservatively. Four of these 12 cases were treated with isotretinoin; however, only one case resulted in any clinical improvement [11,12,16-18]. The patient, in this case, received an initial treatment of cryotherapy twice for five seconds to all lesions with a resultant unsatisfactory response [18]. The patient was then treated with six months of topical tretinoin and 20 mg of systemic isotretinoin with mild to moderate flattening of the lesions [18]. Interestingly, our case’s clinical course ended with the improvement of the patient’s lesions without intervention. Overall, it would appear that even though isotretinoin is the most commonly considered treatment for eruptive syringoma, dermatologists may be overtreating these cases due to patient preference on cosmesis. Particularly for pediatric patients, the pressure to treat may result from parental preference. Due to isotretinoin’s side effects (dry lips - 100%, xerosis - 95.0%, facial erythema - 66.2%, psychiatric symptoms - 25.2%, and eye lesions - 9.0%) [19], treating patients with systemic isotretinoin may be unnecessary if clinical improvement is purely cosmetic and if using isotretinoin is hardly a guarantee for clinical improvement.

## Conclusions

Eruptive syringoma is a rare variant of syringoma, and our case was a classic presentation based on gross and histopathologic examination. This case was particularly significant due to the patient’s young age, ethnicity, and clinical improvement in the absence of treatment. When considering a patient who presents with numerous flesh-colored papules spreading over the periorbital area, neck, chest, abdomen, or axillae, it is important to keep eruptive syringoma in the differential diagnosis. Keeping patient presentation and preference in mind, conservative management should always be considered for these cases.
